# Loot

**Published:** 1872-04

**Authors:** 


### Loot.

***On Arterial Transfusion of Blood.***

Professor Hueter recorded some time ago a case of poisoning with carbonic oxide, in which he preserved the life of the patient by transfusion. More recently, in the *Centralblatt*, he has recommended the same, on the ground of the successful issue of three cases where healthy blood was injected to remove the symptoms accompanying intense septicæmia. Hueter pursued in these cases not the usual plan of injecting venous blood into a vein, but that of injecting the venous blood of a healthy man into the *artery* of the invalid. He performs the operation in the following way: During the defibrination of the blood by beating and filtration through a piece of fine muslin by assistants, he exposes the radial artery or the tibialis posticus, above the malleolus internus, the latter being just as easy to find as the former. Any slight hæmorrhage is carefully arrested, and a very small opening is made in the sheath of the artery, which is separated from the adventitia; a sound is then pushed under the artery, and moved hither and thither, till about two and a half centimetres of the artery are isolated. Four pieces of strong silk are now passed beneath the vessel, of which one forms a reserve ligature. The silk nearest to the heart is now tied tightly, so as to prevent all entrance into the vessel of bloo coming from the heart. The injection syringe is now filled, and the lowermost silk slightly pulled, so as to stretch the vessel. An opening is now made in its upper part, by cutting it about half through transversely with scissors. The canula is introduced, and secured by the third thread. The tension hitherto kept up on the lowermost silk is now relaxed, and the injection begun to be forced in. When the injection is completed, the lowermost thread is tightened, and the piece of artery between the two ligatures excised. The wound is simply dressed. The principal difficulties of the operation are its complexity, and the necessity that exists of maintaining a considerable pressure on the piston to overcome the cardiac pressure. On the latter ground Hueter recommends the employment of Mosler's injection syringe, in which the piston works with a screw.

In Hueter's opinion, the objections are far outweighed by the advantages which arterial transfusion affords. One of these advantages is, that the blood reaches the heart more slowly, and with greater steadiness and regularity, than by venous transfusion. He regards the injection of small quantities (two, three or four ounces) as useless; in most instances from eight ounces to one pound being requisite. But if so large a quantity be suddenly thrown upon the heart, as occurs in injection by the veins, a fatal arrest of its activity may occur. It must be remembered, also, that in consequence of the bleeding prior to the injection, as much unhealthy blood is removed as good is introduced from the system. Another advantage attendant on the arterial injection is security against the introduction of air; any small quantities that may be introduced being rapidly absorbed during the passage of the blood through the capillaries. By this method, also, all danger of phthisis is avoided, which, in many instances, when transfusion of the veins would otherwise have proved successful, has led to the death of the patient.

It has not yet been ascertained whether the contact of a large quantity of blood, rendered arterial by whipping, with the waste of the right heart, is of any real advantage. In the mode of transfusion by the arteries, the blood necessarily becomes venous during its passage through the capillaries.

In conclusion, Prof. Hueter observes that transfusion, whether performed through the veins or arteries, constitutes a weapon against diseases which can in no other way be contested, and points out the excellent results we may anticipate from its employment.—(*Aerztliches Literaturblatt*, No. 6, 1871, and *Langenbeck's Archiv.*, 1870.)—*Practitioner*, Nov., 1871.

### *Treatment of Small-Pox.*

Dr. Stokes read a communication to the Dublin College of Physicians on the treatment of small-pox, especially considered with a view to the prevention of pitting after the disease. He pointed out that the existence of the present epidemic was a sufficient answer to the assumption that small-pox had been stamped out in Ireland. In his opinion another example of the change of type in disease—an asthenic form of zymotic affections having taken the place of their previously existing asthenic character—was afforded in the instance of variola. He believed that the liability to pitting was greater in asthenic than in typhoid cases, and in searching for a reason he had found that the tendency to pit depended mainly on the degree of vascularity of the skin. The indications to be fulfilled in the attempt to check or even entirely to prevent the occurrence of this unpleasant consequence of small-pox, were three: 1, The exclusion of air; 2, the maintenance of the parts affected in a moist state; and 3, the alleviation of local irritation. All these indications were satisfied by the repeated application of soft linseed-meal poultices to the face, which was the part most exposed to the air, consequently the most dry, and lastly the most highly vascular.

Dr. Stokes cited a remarkable case which exemplified the good effect of local depletion of the face by leeches, in preventing the development of pustules and the formation of pits. A young girl was admitted to hospital with every symptom of most intense pyrexia, including terrible headache. So urgent was the last-named symptom, that leeches were applied to the temples in considerable numbers. Two days afterward a dense crop of small-pox vesicles sprang up all over the body and extremities, and soon became confluent, while on the face and neck only a few aborted pustules made their appearance.

If it were required to carry out the indications already alluded to in the case of the entire body, the treatment proposed by Hebra, of continued immersion in a warm bath, was invaluable. In the Meath Hospital a patient at death's door in small-pox, his body being a mass of foul, fœtid and reeking sores, was plunged into a bath and kept there for seven consecutive hours. All delirium ceased at once, the sufferer's torture was at an end, and he quickly became convalescent.

He concluded by giving his hearty testimony of approval of the method introduced by Hebra, pointing out its freedom from danger, the possibility of administering nourishment and stimulants to patients while in the bath, and the fact that the relief given in the most severe confluent cases was at once immediate and permanent.—*Philadelphia Reporter*.

### *The Bavarian Method of Treating Fractures.*

Dr. Corley describes the Bavarian method of treating fractures. The advantages of this plan were plainly these:

The necessity for splints was dispensed with; daily inspection of the injured limb was permitted; the period during which the patient was confined to bed was considerably shortened; and the apparatus was very easily applied, and still more readily taken off. The method was carried out in the following way: A flannel roller, consisting of two pieces of flannel of the length of the affected limb, and stitched together posteriorly, was first placed beneath the limb, in such a way as to bring the line of stitching into correspondence with the middle line of the extremity. It was then carried round the limb on each side and secured in front with long pins having their heads bent at right angles. Over the external surface of this first roller a mixture of plaster of Paris, in equal proportions, was rapidly smeared. A second similar flannel roller was then fastened over all, and secured by a modification of the ordinary many-tailed bandage. Dr. Corley had used this plan in twenty cases, including fractures of the upper and lower extremities, Potts's luxation, Colles's fracture, etc.

***Women of India.***

I shall be under no liability to exaggerate, (meaning, that to exaggerate the wretchedness of woman's condition there were impossible,) nor shall I make a single unsupported statement as to her condition.

The most ancient of human laws now extant, is the Institutes of Menu—the offspring of despotism and priestcraft—which has fixed the social condition of women in India from a thousand years before the birth of Christ; or four thousand years past.

1. Female Infanticide. Girls are not desired, not welcome. Ask a Hindoo, with six children, how many he has; and, if three are boys and three girls, he will answer, three. Seventy-seven per cent. of the females born in one tribe, by their own confession, had been murdered. In another, every girl born in their village, by their own confession, had been destroyed; and by whom was this work done? Generally, by the mother herself. Professing to open the fountain of life to her babe, she coolly and deliberately impregnates it with the elements of death; by putting opium on the nipple of the breast, the child, inhaling it with its milk, dies. These murders are sanctioned by the piety of Hindooism. How apt is the declaration of the apostle: "without natural affection."

Sometimes, a poor, sickly child is put into a basket, carried into the woods and suspended upon the limb of a tree; where, after three or four days, if the vultures, or ants, or beasts of prey have not devoured it, it is supposed the demon which caused the sickness has left it, and if alive, it is taken home.

Marriage and betrothal, with the Hindoos, mean the same thing. This takes place when they are children. The Hindoo Shasters say that a girl is marriageable when she is seven years of age, but she may wait till she is ten.

As soon as a little girl has reached her fifth birth day, her parents begin earnestly to seek a marriage settlement for her. The girl has no choice or voice in her destiny. She is never allowed to see or converse with the man who is to control her. Their law-giver Menu lays the duty upon the father of marrying his daughter before she is twelve years old, under awful penalties—like the guilt of murdering a Brahmin, which is the highest crime. In selecting a wife, Menu says, "She who is not descended from his paternal or maternal ancestors within the sixth degree, and who is not known by the family name to be of the same stock with his father or mother, is eligible by a twice born man" for nuptials and holy union. The phrase "twice born" refers to high caste men.

Then, as to families outside the pale of selection, Menu ordains, "In connecting himself with a wife, let him studiously avoid the ten following families, be they ever so great, or ever so rich in kine, goats, sheep, grain and gold: the family that has omitted prescribed acts of religion; that which has produced no male children; that in which the Veda has not been read; that which has thick hair on the body; and those who have been subject to hemorrhoids, to consumption, to dyspepsia, to epilepsy, to leprosy, and to elephantiasis."

In this, Menu was not so bad, for, many children are born of diseased parents in Christian lands which might be avoided.

"A Brahmin should, according to law, marry a maiden about a third of his own age;" and "a Hindoo bride should not be over twelve years of age."

The chief thing to settle upon is her name, for wives there do not take the names of their husbands as they do here. There the wife is ever known only by her maiden name; hence the name is of vital importance, and the law generally prescribes as follows: "Let him not marry a girl with the name of a constellation, of a tree, of a river, of a barbarous nation, or of a mountain, or of a winged creature, snake, or a glove; nor with any name raising an image of terror. The names of women should be agreeable, soft, clear, captivating the fancy, auspicious, ending in long vowels, resembling words of benedictions."—*Rev. William Butler, D.D.* "*Land of the Veda.*"

### *Curiosities of Corns.*

A curious fact is related of the Maine wood-choppers, who, in the winter months, while felling trees amid snow and ice, cannot wear close-fitting boots, but very large sizes, that allow their feet to slip to and fro, thus causing friction and warmth. But from this they have feet completely encrusted with corns. In the spring, when the raft goes down with their logs, they do not change their clothing or boots for weeks, until their arrival at the saw-mills. All the while, their feet have been saturated in water, and when the boots are removed, the corns fall off like nutshells from the kernel.—*Haney's Journal.*

### *How Crude Rubber is Collected.*

Greytown, Nicaragua, is the principal port for the export of india-rubber on the coast. It is collected by parties of Indians, Caribs, or half-caste Creoles—seldom by Europeans—to whom the dealers, who are also storekeepers, advance the necessary outfit of food, clothing, and apparatus for collecting rubber, on condition of receiving the whole of the rubber collected at a certain rate. The rubber-hunters are termed *Uleros* (*Ule* being the Creole term for rubber).

A party of Uleros, after a final debauch at Greytown, having expended all their remaining cash, generally make a start in a canoe for one of the rivers or streams which abound on the coast, and having fixed on a convenient spot for a camp, commence operations. The experienced rubber-hunter marks out all the trees in the neighborhood. The rubber-tree is the *Castilloa elastica*, which grows to a great size, being on an average about four feet in diameter, and from twenty to thirty feet to the first spring of the branches. From all the trees in the almost impenetrable jungle hang numerous trailing parasites, lianes, etc.; from these, and especially the tough vines, are made rude ladders, which are suspended close to the trunks of the trees selected, which are now slashed by machetes in diagonal cuts from right to left, so as to meet in the middle and central channels, which lead into iron gutters driven in below, and these again into the wooden pails. The pails are soon full of the white milk, and are emptied into larger tin pans. The milk is next pressed through a sieve, and subsequently coagulated by a judicious application of the juice of a Bejuca (an *Apocyna*) vine. The coagulated mass is then pressed by hand, and finally rolled out on a board with a wooden roller. The rubber has now assumed the form of a large pancake, nearly two feet in diameter, and about a quarter of an inch thick, on account of which they are termed *tortillas* by the Uleros; these cakes are hung over the side poles and framework which supports the *rancho*, which is erected in the woods, and allowed to dry for about a fortnight, when they are ready to be packed for delivery to the dealer.—*Scientific American.*

***Lightning Stroke.***

Dr. C. H. Alden, U. S. A., reports (Circular No. 3) a case, of which the following contains the essential outlines. E. W., aged 60, was struck whilst on horseback, and very shortly afterwards brought in. The horse was killed. The man was totally insensible but very restless, tossing, and resisting efforts to remove his wet clothing. His surface and extremities were cold, his pulse very small and feeble. There was a superficial, nearly vertical wound of the right scalp, looking as though made with a sharp point; a chain of large, irregular vesicated spots extended down the right ear over the neck, chest and abdomen, to the penis. There was also a similar spot on the right thigh, and superficial abrasions on both thighs. His felt hat was much torn and burnt on the right side, his pantaloons burnt corresponding to the thigh wounds; his shirt somewhat torn in front. He was wrapped in blankets, and whisky administered, which he swallowed without difficulty. In the afternoon slight vomiting occurred, and his pulse grew stronger and fuller. During the evening and much of the night, he was restless, muttering incoherently, and having involuntary passages. The next day his condition was much the same, but he was quiet a good deal of the time, and his pulse fuller, surface warmer; a little fluid trickled from the right ear. Weak milk punch and beef-tea were ordered. In the afternoon he opened his eyes, looked round intelligently, but did not speak, and in a few minutes became again unconscious; at 7.30 P. M. pulse 72; respirations, 30; temperature, 97°. He died at midnight. Post mortem rigidity came on in five hours.

At the autopsy slight bloody infiltration of the muscles, below the scalp wound, was found. A very slight fissure was found inside calvarium, corresponding to the external wound. Between the bone and the dura mater was a firm, black, circular clot, one-fourth of an inch in thickness, and two inches in diameter; opposite the centre of the clot was a minute opening in the dura mater. The brain beneath the clot was disorganized for about two inches in diameter, and extending into the ventricle. Nothing else abnormal was anywhere discovered.

---

### *American Medical Association.*

The Twenty-third Annual Session will be held at Philadelphia, in Horticultural Hall, Broad street, above Spruce, on Tuesday, May 7, 1872, at 11 A. M.

HOTEL ARRANGEMENTS.

Continental, Chestnut and 9th, $4 a day.
Girard, Chestnut and 9th, $3 a day.
La Pierre, Broad below Chestnut, $3 a day.
Colonnade, Chestnut and 15th, $3 a day.
St. Cloud, Arch below 8th, $3 a day.
St. Elmo, Arch above 3rd, $2.50 a day.
American, Chestnut below 6th, $2.50 a day.
Merchants, 4th above Market, $2.50 a day.
St. Lawrence, Chestnut below 12th, $2 a day.
Alleghany, Market below 9th, $1.75 a day.
St. Charles, 3rd below Arch, lodging only, 50 cents a day.
Miller's, 7th and Chestnut, lodging only, $1.50 a day.
Meals at restaurant of Horticultural Hall, and Petry's, N. W. cor. Broad and Walnut, each 50 cents.

BOARDING HOUSES.

318 South Broad, $2 a day; or $10 a week.
N. E. cor. Broad and Spruce, $1.50 a day; or $10 a week.
329 South Broad, $2 a day; or $10 a week.
1327 Spruce street, $2 a day; or $12 a week.
225 South Broad, $2.50 a day; or $12 a week.

RAILROADS.

Union Pacific, return free, if first-class tickets are bought, and an acknowledgment taken from the agent.
Cumberland Valley, excursion tickets.
Orange, Alexandria and Manassas, half fare for return.
Pittsburgh, Cincinnati and St. Louis, excursion tickets.
Pittsburgh, Fort Wayne and Chicago, excursion tickets.
Cleveland and Pittsburgh, excursion tickets.
Central Railroad of Georgia, return free.
Richmond and Petersburg, return free.
Wilmington and Weldon, excursion tickets, one fare.
Wilmington, Columbia and Augusta, excursion tickets, one fare.
Kansas Pacific, one and one-fifth fare for excursion.
Atlanta and New Orleans Short Line (A. and W. Pt. Western, Mobile and M. N. O. M., and Texas Railroads), return free.
Western and Atlantic, excursion tickets, one fare.
Western Alabama, excursion tickets, one fare.
Evansville and Crawfordsville, excursion tickets.
Lehigh Valley, excursion tickets, one fare.
Louisville and Nashville, excursion tickets.
Memphis and Louisville, excursion tickets.
North Pennsylvania, excursion tickets, two-thirds fare.
Pennsylvania Central, excursion tickets.
Philadelphia and Erie, excursion tickets.
Philadelphia, Wilmington and Baltimore, excursion tickets.
Philadelphia and Reading, excursion tickets at two-thirds.
Baltimore and Ohio, excursion tickets.
Lake Shore and Michigan Southern, excursion tickets if forty are taken.

WM. B. ATKINSON, *Per. Sec.*

